# Structural basis of purine nucleotide inhibition of human uncoupling protein 1

**DOI:** 10.1126/sciadv.adh4251

**Published:** 2023-05-31

**Authors:** Scott A. Jones, Prerana Gogoi, Jonathan J. Ruprecht, Martin S. King, Yang Lee, Thomas Zögg, Els Pardon, Deepak Chand, Stefan Steimle, Danielle M. Copeman, Camila A. Cotrim, Jan Steyaert, Paul G. Crichton, Vera Moiseenkova-Bell, Edmund R. S. Kunji

**Affiliations:** ^1^ MRC Mitochondrial Biology Unit, University of Cambridge, Cambridge Biomedical Campus, Keith Peters Building, Cambridge CB2 0XY, UK.; ^2^ Department of Systems Pharmacology and Translational Therapeutics, University of Pennsylvania, Perelman School of Medicine, 10-124 Smilow Center for Translational Research, 3400 Civic Center Boulevard, Philadelphia, PA 19104-5158, USA.; ^3^ VIB-VUB Center for Structural Biology, VIB, Pleinlaan 2, B-1050 Brussels, Belgium.; ^4^ Structural Biology Brussels, Vrije Universiteit Brussel, Pleinlaan 2, B-1050 Brussels, Belgium.; ^5^ Department of Biochemistry and Biophysics, University of Pennsylvania, Perelman School of Medicine, Philadelphia, PA 19104, USA.; ^6^ Biomedical Research Centre, Norwich Medical School, University of East Anglia, Norwich NR4 7TJ, UK.

## Abstract

Mitochondrial uncoupling protein 1 (UCP1) gives brown adipose tissue of mammals its specialized ability to burn calories as heat for thermoregulation. When activated by fatty acids, UCP1 catalyzes the leak of protons across the mitochondrial inner membrane, short-circuiting the mitochondrion to generate heat, bypassing ATP synthesis. In contrast, purine nucleotides bind and inhibit UCP1, regulating proton leak by a molecular mechanism that is unclear. We present the cryo–electron microscopy structure of the GTP-inhibited state of UCP1, which is consistent with its nonconducting state. The purine nucleotide cross-links the transmembrane helices of UCP1 with an extensive interaction network. Our results provide a structural basis for understanding the specificity and pH dependency of the regulatory mechanism. UCP1 has retained all of the key functional and structural features required for a mitochondrial carrier–like transport mechanism. The analysis shows that inhibitor binding prevents the conformational changes that UCP1 uses to facilitate proton leak.

## Teaser

Purine nucleotides inhibit uncoupling protein 1 in a proton-impermeable intermediary state by a pH-dependent mechanism.

## INTRODUCTION

Uncoupling protein 1 (UCP1) is a 33-kDa mitochondrial carrier protein that gives brown and beige adipose tissue of mammals its unique ability to burn off cellular nutrients as heat in the process of nonshivering thermogenesis, helping to protect the body against cold temperatures (*1*–*3*). When activated, the protein catalyzes proton leak across the mitochondrial inner membrane, dissipating the proton motive force that would otherwise drive adenosine triphosphate (ATP) synthesis, to release energy as heat. Thermogenesis via UCP1 acts as a potent sink for disposal of blood glucose and triglycerides, and has the potential to combat obesity and metabolic disease and to restrict the growth of glucose-dependent cancers (*1*–*3*). Active human brown/beige fat correlates with improved insulin sensitivity and glucose homeostasis (*4*–*6*) and inversely with age-related obesity in the population (*7*–*10*). To engage thermogenesis, UCP1 is activated by free fatty acids, released by intracellular lipolysis following upstream adrenergic stimulation of brown adipocytes (e.g., in response to cold exposure). Fatty acids interact directly with UCP1 to overcome the binding and inhibition of the protein by cytosolic purine nucleotides, but the mechanism is unresolved (*11*). Pharmacological intervention to promote brown fat and UCP1 activation, without the need for physiological stimuli, are a therapeutic strategy to combat metabolic disease (*12*). The nature of UCP1 proton leak and regulation by ligands has been debated for many years, confounded by a lack of structural information. Several biochemical models have been proposed to explain UCP1 activity in relation to its regulators, each based largely on observations from different experimental systems (*13*). Fatty acids may act to remove nucleotide inhibition of UCP1, either by direct competition or allosterically, to leave an intrinsically active protein (competition model) (*14*), or act as an essential cofactor, providing a key protonatable carboxylate to complete a proton conductance pathway in UCP1 (cofactor model) (*15*). Alternatively, fatty acids may act as a transport substrate of UCP1. The export of fatty acid anions by the protein may occur, where, after protonation, the fatty acid flips back across the membrane independently of UCP1 (cycling model) (*16*, *17*), or the fatty acid anion may remain bound and return via UCP1 chaperoning the proton (shuttling model) (*18*), in either case, to give a net proton transfer.

UCP1 (*SLC25A7*) is a member of the solute carrier family 25 (SLC25), which all share a common structural fold (*19*). Carriers cycle between cytoplasmic-open and matrix-open states (c- and m-states) to alternate access of a substrate binding site within the central cavity of the protein to either side of the membrane for stepwise substrate exchange. Crystal structures of the mitochondrial ADP/ATP carrier in a c-state (*20*, *21*) and an m-state (*22*) have revealed the molecular mechanism of transport (*23*). The conformational changes are complex, involving three core elements and three gate elements, which move separately, but in a coordinated way around a central substrate translocation pathway (*22*). UCP1 was previously thought to exist and function as a dimer (*24*), but native UCP1 was shown to be monomeric and bind cardiolipin, as other carriers (*25*). Ligand-binding analyses indicate that activators destabilize UCP1, consistent with potential state shifts associated with transport (*26*). In contrast, purine nucleotides, which bind one nucleotide per UCP1 monomer (*25*), stabilize the protein (*27*), particularly at pH values below 6.5, where the affinity is the highest (*28*). However, the molecular details of how proton leak occurs and how regulators bind to control the activity of UCP1 remain unresolved. Here, we present the structure of the human UCP1 inhibited by guanosine triphosphate (GTP), solved by cryo–electron microscopy (cryo-EM). The details reveal how nucleotides interact to inhibit conformational changes in UCP1, which underlie the proton conductance mechanism.

## RESULTS

### Guanine nucleotide inhibition and proton conductance of human UCP1

**Fig. 1.**
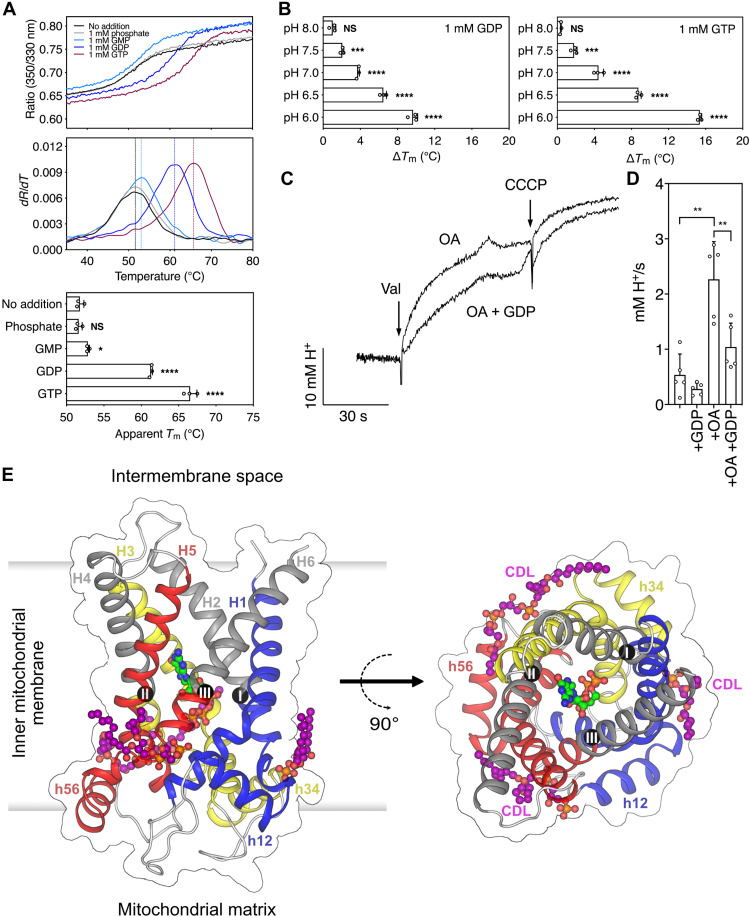
Functional and structural properties of human UCP1. (**A**) Binding of guanine nucleotides to human UCP1 measured by thermal stability shift assays. Representative unfolding curves of UCP1 (black trace), incubated with 1 mM phosphate (gray trace), guanosine monophosphate (GMP; light blue trace), guanosine diphosphate (GDP; blue trace), or GTP (purple trace) at pH 6. The peak in the derivative of the unfolding curve (*dR*/*dT*) is the apparent melting temperature (*T*_m_, dashed line). (**B**) pH dependence of the thermal shift (∆*T*_m_) by guanosine di- and trinucleotides (1 mM). The individual unfolding traces are shown in fig. S1. The average and SD of three biological repeats. Two-tailed Student’s *t* tests assuming unequal variances [0.05 < *P*, not significant (NS); *0.01 < *P* < 0.05; **0.001 < *P* < 0.01; ***0.0001 < *P* < 0.001; ****P < 0.0001]. (**C**) Proton uptake activity by reconstituted UCP1 induced by the generation of a membrane potential with valinomycin (Val) followed later by carbonyl cyanide 3-chlorophenylhydrazone (CCCP), as a chemical uncoupler control in the absence or presence of 100 μM oleic acid (OA) and 1 mM GDP. (**D**) Average initial rates in the presence of the activator oleic acid and inhibitor GDP. The average and SD of three to five biological repeats, as indicated. (**E**) Lateral and cytoplasmic view of the structure of UCP1 with bound GTP (green) and three cardiolipin molecules (purple). Core elements 1, 2, and 3 are colored by domain in blue, yellow, and red, respectively, and the gate elements in gray. The contact points are shown as black spheres with Roman numerals.

We expressed human UCP1 in yeast mitochondria and validated its functional properties following purification. UCP1 was tested for its ability to bind guanine nucleotides by using thermostability shift assays with differential scanning fluorimetry (nanoDSF) (*29*), which can be used to study the interactions of inhibitors, ligands, and substrates (*27*, *30*–*32*). In the absence of ligand, detergent-solubilized UCP1 produced an unfolding curve with an apparent melting temperature (*T*_m_) of 52°C (Fig. 1A), similar to other SLC25 mitochondrial carriers (*27*, *30*–*32*). At pH 6.0, guanine nucleotides, but not a phosphate control, induced a significant increase in UCP1 stability, particularly the tri- and diphosphate purine nucleotides, GTP (*T*_m_, 67°C) and guanosine diphosphate (GDP) (*T*_m_, 61°C), which are known to bind with higher affinity than guanosine monophosphate (GMP) (*28*). The shift induced by GTP and GDP (~15°C and ~10°C, respectively) was highly sensitive to pH, decreasing incrementally with a rise in pH from 6.0 to 8.0 (Fig. 1B and fig. S1), consistent with the characteristic drop in purine nucleotide affinity observed for rodent and ovine UCP1 (*27*, *28*). When the unliganded protein was reconstituted into liposomes for activity assessment, human UCP1 catalyzed proton conductance that was significantly activated by oleic acid and inhibited by GDP (Fig. 1, C and D), confirming that recombinantly expressed human UCP1 has the expected properties of the native protein.

### Structure of the GTP-bound UCP1 in complex with two Pro-macrobodies

To obtain the structure, UCP1 was stabilized by GTP, the nucleotide that binds with highest affinity (*28*). We also selected nanobody 65 and 71, which stabilized UCP1 by 5.0° and 0.8°C, respectively, which is far less than the stabilisation by GTP (fig. S2). Next, we generated two Pro-macrobodies (PMb65 and PMb71), which are nanobodies (*33*) fused with maltose-binding protein (MBP) via a Pro-Pro linker (*34*). In this way, the size, stability, and asymmetry of the protein complex were increased, allowing image data to be collected by cryo-EM and analyzed by single-particle analysis (fig. S3 and table S1). The resulting density map (fig. S4) was of sufficient quality to build the structure of human UCP1 with one GTP molecule and three (incomplete) cardiolipin molecules (Fig. 1E). The map also contained electron densities for PMb71 with bound maltose and regions of PMb65, which bind to the matrix and cytoplasmic side of UCP1, respectively (fig. S4, A and B). PMb65 interacts with the cytosolic loop between H4 and H5, whereas PMb71 interacts with two matrix loops of UCP1 (fig. S5).

UCP1 has a monomeric structural fold and structural features expected of a SLC25 member (*35*, *36*), consistent with our composition and sequence analysis (Fig. 1E) (*13*, *25*). Similar to the related ADP/ATP carrier (*20*, *21*), UCP1 has three homologous domains, arranged threefold pseudo-symmetrically (fig. S6). The domains consist of two transmembrane helices linked by a loop and small matrix helix (h12, h34, or h56). Together, the six transmembrane helices (H1 to H6) surround a central water-filled cavity (Fig. 1E). Helices H1, H3, and H5 have an L-shape due to highly conserved proline residues at the kink (*20*). The central cavity of GTP-inhibited UCP1 is open to the intermembrane space, whereas the matrix side is closed (Fig. 1E), revealing a c-state–like conformation that resembles the carboxyatractyloside-inhibited structure of the ADP/ATP carrier (fig. S6) (*20*, *21*). However, the transmembrane helical bundle of UCP1 is much tighter on the intermembrane space side, and, consequently, the water-filled cavity is narrower (fig. S6). The structure confirms that three cardiolipin molecules are bound to UCP1 (*25*), unlike previous claims (fig. S7A) (*24*). The negatively charged phosphate head groups are bound to the N termini of the matrix helices and even-numbered helices by hydrogen bonds and electrostatic interactions with the helix dipoles (fig. S7B), giving positively charged patches (fig. S7C). These sites are found in loop-to-helix transitions, which contain highly conserved glycine or serine residues, which act as helix breakers (fig. S7D).

### Proton-impermeable state of UCP1 due to nucleotide binding

**Fig. 2.**
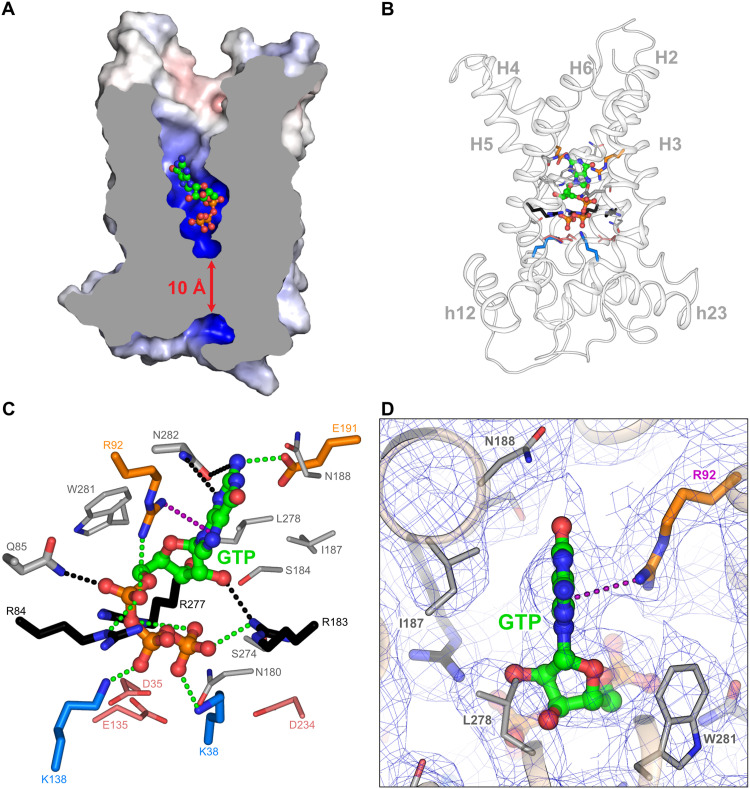
GTP binding site of the human UCP1. (**A**) Cross section through UCP1 showing the central water-filled cavity with GTP (green). The surface is colored by the electrostatic potential (blue, +15 kT *e*^−1^; white, neutral; red, −15 kT *e*^−1^), calculated by ABPS. (**B**) Lateral overview and (**C**) details of the GTP (green) binding site. The arginine triplet of the contact points is shown in black; the positively and negatively charged matrix network residues are shown in blue and red, respectively; the cytoplasmic insulator residues R92 and E191 in orange; and the rest in gray. Residues in the site that have no direct interactions are shown as thin sticks. Salt bridge, hydrogen bond, and cation-π interactions are shown in green, black, and purple dotted lines, respectively. (**D**) Detail of the cryo-EM map, contoured at 12.5 root mean square deviations above the mean, showing the cation-π interaction of R92 with the guanine base of GTP.

GTP binds deeply in the central cavity of UCP1, with the triphosphate moieties positioned to interact with the positive electrostatic potential at the base, generated by R84, R183, and R277 (the arginine triplet) (Fig. 2, A and B). GTP forms salt bridge and hydrogen bond interactions with the side chains of multiple residues, many of which are functionally important (Fig. 2, B and C, and fig. S8). The ɣ-phosphate of GTP forms salt bridges to K38, part of the matrix gate network, and to R183 and R277, which are contact points of the substrate binding site in other mitochondrial carriers (*23*). The β-phosphate of GTP forms a salt bridge to K138, another residue of the matrix gate network. The α-phosphate of GTP forms a salt bridge to R84, a putative substrate contact point, and a hydrogen bond to Q85. The GTP ribose group forms a hydrogen bond to R183. At the cytoplasmic end of the binding site, the guanine group forms hydrogen bonds to N282 and a salt bridge to E191, and the guanine ring forms a cation-π interaction with R92. A redistribution of the electrostatic potential distribution due to the cation-π interaction is visible in the map (Fig. 2D). The considerable interactions with the nucleotide constrain UCP1 in a pose with an impermeable barrier at the matrix side, explaining why proton leak cannot occur (Fig. 2A).

The observed interactions explain differences in the experimentally determined stability of UCP1 inhibited by guanine nucleotides (Fig. 1A). The increasing thermal stability from GMP to GTP correlates with the increasing number of interactions from α- to ɣ-phosphate. In agreement, mutagenesis of R84, R183, R277, and R92 and chemical modification of E191, a residue identified to play a role in pH control, abolish nucleotide binding (*37*, *38*).

### Closing of the cytoplasmic gate and opening of the matrix gate prevented by nucleotide binding

**Fig. 3.**
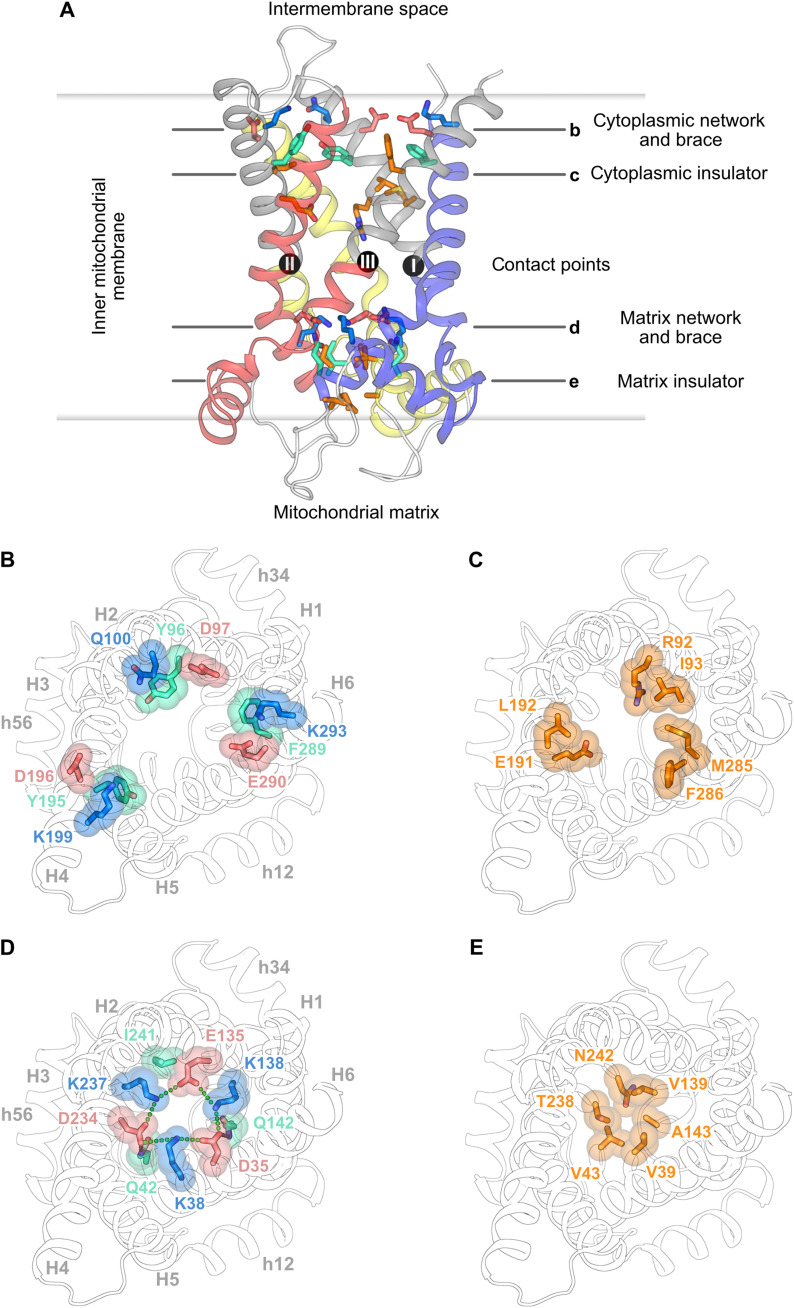
Structural properties of the matrix and cytoplasmic gate of human UCP1. (**A**) Lateral view of UCP1 with the salt bridge network, braces, and insulator layers. Cytoplasmic view of (**B**) cytoplasmic salt bridge network and tyrosine braces, (**C**) cytoplasmic insulator, (**D**) matrix salt bridge network and glutamine braces, (**E**) matrix insulator. Helices and contact points are shown as in Fig. 1E.

The lack of a proton conductance pathway in the GTP-bound structure of UCP1 indicates that activation involves conformational state changes in the protein, consistent with proteolytic studies and recent activator binding analysis (*26*). The structure of UCP1 confirms the presence of key structural features of a carrier-like mechanism, such as the presence of cytoplasmic and matrix gates (Fig. 3A) (*22*).

The cytoplasmic gate is open, and the residues of the cytoplasmic salt bridge network (D97, Q100, D196, K199, E290, and K293), the tyrosine braces (Y96 and Y195) (Fig. 3B), and the cytoplasmic insulation layer (Fig. 3C) are not engaged. These residues are located on the gate elements of H2, H4, and H6, and they form several interactions with GTP, such as with R92, N188, E191, and N282, preventing closure of the gate.

**Fig. 4.**
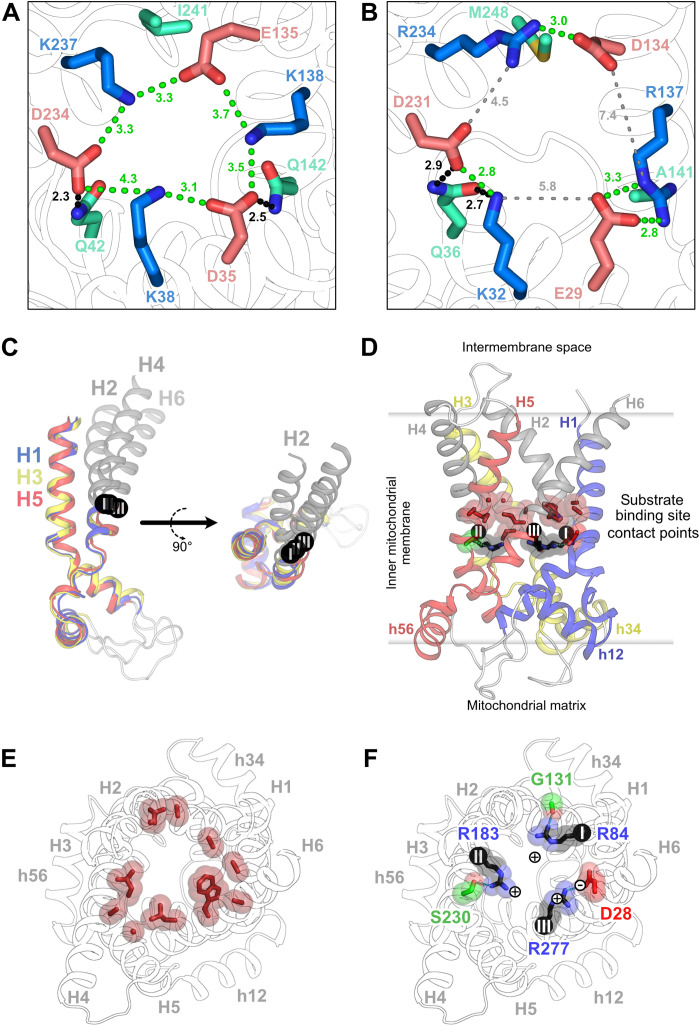
Matrix salt bridge network and binding site region of human UCP1. (**A** and **B**) Matrix salt bridge network and glutamine braces of human UCP1 and bovine ADP/ATP carrier, respectively. (**C**) Domain structures of UCP1, superimposed on the core elements. (**D**) Lateral view of UCP1, showing the location of the contact points and substrate binding site region. (**E** and **F**) Detailed cytoplasmic view of hydrophobic and contact point residues (arginine triplet), respectively. Helices and contact points colored as in Fig. 1E.

The matrix gate (Fig. 3A) is clearly closed, forming an insulation layer of ~10 Å to prevent the conductance of protons (Fig. 2A). A key component is the matrix salt bridge network on H1, H3, and H5 (D35, K38, E135, K138, D234, and K237) (Fig. 3D), which forms ionic interactions between the core elements (Fig. 4A). Glutamine residues Q42 and Q142 function as braces by hydrogen bonding to D234 and D35, respectively (Figs. 3D and 4A). The closing of the matrix gate is further supported by the pronounced kinks in the odd-numbered transmembrane helices that bring their C-terminal ends close together beneath the matrix network (Fig. 3, A and E).

### Key features of the carrier substrate binding region of UCP1

Activating compounds could act as conventional substrates of UCP1. In mitochondrial carriers, substrates are recognized by specific residues on H2, H4, and H6 that act as the main substrate contact points, determining aspects of substrate specificity (*39*, *40*). The same residues were later found to act as hinge points between the core and gate elements, crucial for the structural changes in the transport mechanism (*22*). The corresponding contact points in UCP1 are arginine triplet R84, R183, and R277 (Fig. 4, D and F), which are also found in the phylogenetically related dicarboxylate and oxoglutarate carriers. The structure confirms a dominant position of the triplet in the cavity for potential ligand interactions. The arginine triplet generates a highly positively charged cavity (Fig. 2A), which would attract not only negatively charged compounds, such as the inhibitor GTP, but also fatty acid activators, which can be transported by UCP1 (*18*). Other residues in close proximity would facilitate transport substrate–like interactions (e.g., Q85 and S184) (*41*), including an unexpected number of hydrophobic residues in the water-filled cavity for hydrophobic interactions (e.g., V128, I187, L278, W281, and M285) (Fig. 4, D and E). The positively charged side chains of the arginine triplet are kept away from each other by specific interactions: R84 with the carbonyl backbone group of G131, R183 with S230, and R277 with D28 (Fig. 4, D and F). D28, which is absent in dicarboxylate and oxoglutarate carriers and revealed here to be present in the UCP1 binding site region, has been shown to be required for fatty acid activated proton conductance (*42*).

### Involvement of core and gate elements in GTP binding

To investigate potential conformational changes of GTP-inhibited UCP1, we superimposed the core elements of domains 1, 2, and 3, as previously done for the c- and m-state structures of the ADP/ATP carrier (Fig. 4C) (*22*). This superimposition shows that the structure of the core elements overlays well but that the gate elements are rotated to different degrees. The gate element of H6 is in a position typical for a c-state structure, whereas the gate element of H2 is highly rotated, in a position more akin to that of the m-state. Notably, the helices in the GTP-locked structure of UCP1 are in a tighter configuration than the known c-state of the ADP/ATP carrier (fig. S6), consistent with the UCP1 structure displaying a c-state arrangement partially transitioned toward an m-state in a carrier-like transport mechanism. The hinge points of these movements correspond to the putative contact points of the substrate binding site, arginine triplet R84, R183, and R277 (Fig. 4, C and D), as observed for the ADP/ATP carrier (*22*). Although these different rotations must be influenced by the interactions with GTP, they show that, in principle, UCP1 could use c-state–to–m-state interconversions. Further support for this notion comes from the observation that UCP1 has small amino acid residues (Gly, Ala, Ser, Val, and Cys) in the interhelical surfaces on the cytoplasmic side, which are crucial for the conformational changes of mitochondrial carriers (fig. S9) (*22*, *35*, *36*).

## DISCUSSION

### Key features of the inhibitory function of GTP

Here, we present the structure of UCP1 locked in the cytoplasmic state by GTP, which represents a state that does not conduct protons. The mode of GTP binding to UCP1 reveals five key features important for inhibition of the proton conductance. First, the structure shows that UCP1 has a large insulation layer, preventing the flux of protons, consistent with its nonconducting state (Fig. 2A). Second, GTP occupies the carrier substrate binding site, preventing the binding of activators (Fig. 2B). Third, GTP cross-links the transmembrane helices together by an extensive interaction network, involving ionic, polar, and cation-π interactions (Fig. 2, C and D), explaining the stabilizing effects of the inhibitor (Fig. 1A). Fourth, GTP interacts with the functionally important matrix salt bridge network, preventing it from disrupting, which is required for state interconversion in other SLC25 members (Fig. 2C). Fifth, by binding extensively to the gate elements, which are also important in the transport mechanism, GTP prevents the closure of the cytoplasmic side of UCP1. These features are all consistent with the formation of an abortive state due to binding of the inhibitor, which prevents proton leak. Thus, in all of the key principles, but in none of the chemical detail, GTP binding to UCP1 resembles the way that carboxy-atractyloside interacts with the mitochondrial ADP/ATP carrier, generating an abortive c-state (*20*, *21*).

The binding pose of GTP in the central cavity of UCP1 provides important clues to the specificity of inhibitor binding, as both GTP and ATP are known to bind to UCP1 with subtle differences in affinity (*28*). In agreement, the vast majority of polar interactions are with the ribose and phosphate moieties, which are common to both. Furthermore, the cation-π interaction of R92 with the guanine base of GTP could also occur with the adenine base of ATP (Fig. 2, C and D). There are two asparagine residues: N282 and N188 (Fig. 2 and fig. S8), which are well placed to interact with the N_2_ amine of guanine or the N_6_ amine of adenine, respectively. The small differences in affinities could be explained by the fact that adenine has no functional group to interact with E191, a residue known to influence nucleotide affinity (*38*, *43*).

The pH dependency of purine nucleotide binding to UCP1, showing decreased binding affinity above pH 6.5, is another well-established property (*44*, *45*), confirmed independently here (Fig. 1B). The structure of UCP1 was solved at pH 6, where purine nucleotides bind with high affinity. The equivalent residue of E191 has been thought to be a major reason for the pH dependency of nucleotide binding in rodent UCP1 (*38*, *43*, *44*). As shown here, the negatively charged E191 binds to the delta-positive N_2_ amine of guanine, but this interaction would not be affected by a pH shift around 6.5 [p*K*_a_ (where *K*_a_ is the acid dissociation constant) values of guanine are ~3.3 and ~9.5]. When E191 is replaced with glutamine, UCP1 still binds GTP, but the affinity is reduced (*43*), as explained by the elimination of the electrostatic component. A prominent reduction in the amount of GTP binding at the higher pH of 7.5 (*43*) still occurs in the E191Q mutant, albeit with improved affinity over the wild type protein, and, thus, E191 cannot be the primary reason for the pH effect. GTP has a p*K*_a_ value at 6.5 (*46*), which corresponds well to the pH switch between low and high affinity binding. Below pH 6.5, GTP is protonated, being predominantly GTP^3−^, whereas, above pH 6.5, it is unprotonated, being mostly GTP^4−^. At pH 6, the D35 and E135 residues of the matrix salt bridge network are in bonding distance to the γ-phosphate and β-phosphate of GTP, respectively (Fig. 2C). Interactions of the phosphate moieties with these negatively charged residues could occur by forming proton-mediated hydrogen bonds with protonated GTP at low pH (Fig. 2). At high pH, the phosphate and carboxyl groups would both be negatively charged, leading to repulsion and, consequently, to a highly reduced affinity (Fig. 1B), explaining the pH effect.

### Properties of the substrate binding region consistent with fatty acid and proton binding

Members of the SLC25 mitochondrial carrier family to which UCP1 belongs have a central substrate binding site, which consist of three contact points (*39*, *40*) and a hypervariable site (*41*) that bind the various substrates. This site is located centrally, corresponding to the midpoint of the membrane, because the matrix and cytoplasmic gates need to open and close alternately on either side of this site (*22*, *41*). UCP1 does have both the matrix and the cytoplasmic gate, which contain matrix salt bridge network and glutamine braces and cytoplasmic salt bridge network and tyrosine braces, respectively. Thus, it is of interest to see what properties the region in between the gates has, which corresponds to the common substrate binding site of mitochondrial carriers (*39*–*41*). An unexpectedly large number of hydrophobic residues are present in this area for a water-filled cavity (Fig. 4, D and F). The contact points are a triplet of symmetrically arranged positively charged arginine residues, R84, R183, and R277 (Fig. 4F), which double up as hinges between the core and gate elements (Fig. 4C), which are a key feature of the transport mechanism of mitochondrial carriers. The arginine triplet generates a highly positively charged cavity (Fig. 2A), which could attract not only negatively charged compounds, such as the inhibitor GTP, but also fatty acids. These residues are fixed in position by specific interactions: R84 with the carbonyl backbone group of G131, R183 with S230, and R277 with D28, which has been shown to be involved in proton translocation (*42*). This arrangement suggests that binding of a negatively charged fatty acid activator, as a transport substrate of UCP1 (*16*–*18*), to R277 and proton binding to D28 might release the R277:D28 salt bridge, triggering conformational changes. Several mitochondrial carriers use proton-coupled import of their substrates against the concentration gradient, such as the phosphate, citrate, and GDP/GTP carriers (*47*). They have positively charged residues, which are important for substrate binding, in bonding distance of negatively charged residues important for proton binding (*47*). Furthermore, in a large number of other transport proteins, unrelated in structure and mechanism, a similar arrangement can be observed, such as the lactose permease (*48*, *49*), folate transporter (*50*, *51*), monocarboxylate transporter (*52*, *53*), proton-dependent oligopeptide transporters (*54*), and metal-ion transporters (*55*), indicating a common principle. As such, the central binding site region has properties that are compatible with fatty acid and proton binding, which could occur as part of the activation mechanism.

### Conformational changes as part of the activating mechanism

The overall interdomain bonding arrangement of the matrix network stabilizes the protein in a c-state–like conformation, similar to the equivalent state of the ADP/ATP carrier (*20*, *21*). However, unexpectedly, the matrix network is distorted in UCP1 (Fig. 4A). The alternating positively and negatively changed residues that form the main ionic interdomain interactions of the network do not pair up, as observed in the ADP/ATP carrier (Fig. 4B), but instead maintain both intra- and interdomain interactions, giving an overall ionic bonding “ring” (Fig. 4A). Both K38 and K138 of the matrix network interact directly with GTP (Fig. 2C), and the resulting reorientation of side chains is likely responsible for the observed network changes. Thus, GTP prevents disruption of the matrix network, a prerequisite for conformational change, by cross-linking the matrix network extensively. Furthermore, the superimposition of the domains shows that the structure of the core elements overlays well but that the gate elements are rotated to different degrees. The gate element of H6 is in a position typical for a c-state structure, whereas the gate element of H2 is highly rotated, in a position more akin to that of the m-state. Thus, GTP-inhibited UCP1 is not in a pure cytoplasmic state, as the carboxyatractyloside-inhibited ADP/ATP carrier, but in an intermediary state. Hence, the structural organization is consistent with UCP1 undergoing transitions between the c- and m-states as part of its mechanism. Nucleotide binding prevents this transition by occupying the central binding site, by cross-linking the matrix network and preventing it from disrupting, and by blocking the movement of the gate elements, preventing the formation of the cytoplasmic gate. Substrate binding at the key contact points could initiate transition to an m-state, facilitating proton translocation in a mechanism that could involve D28. Fatty acid activators, which induce destabilizing shifts in purified UCP1 akin to substrate binding in other carriers (*26*), likely bind as transport substrates to drive state changes, using an m-state configuration as part of the proton conductance mechanism. The molecular details elucidated here provide a crucial framework for understanding UCP1 and related carriers (UCP1 to UCP5) and for developing interventions to target these important proteins for better health (*12*, *56*), such as the development of compounds or state-specific nanobodies for therapeutic purposes.

## MATERIALS AND METHODS

### Expression and purification of wild-type human UCP1

Where required, the gene for wild-type human UCP1 (UniProt accession code P25874) was modified to encode an upstream N-terminal His_8_ tag and Factor Xa protease cleavage site, using the following primers: forward primer: GAATTCGAGCTCAAAAAATGCATCATCACCATCACCATCATCATGATGCAGCAATTGAAGGTAGGACATCTGAAGATGGAGGATTAACAGCATCAGACGTTCACCCTACT; reverse primer: GGTACCCTCGAGCTATCATTATGTAGCACAGTCCATTGTTTGTCTTGATTTACTAAG.

Expression vectors pYES2/CT or pYES3/CT were electroporated into *Saccharomyces cerevisiae* strain W303-1B (*MAT*α *leu2-3,112 trp1-1 can1-100 ura3-1 ade2-1 his3-11,15*). Positive transformants were selected on Sc-Ura+glucose plates. Cells containing the desired construct were grown in 2 liters of preculture medium [0.67% Yeast Nitrogen Base, 0.1% KH_2_PO_4_, 0.12% (NH_4_)_2_SO_4_, 0.1% casamino acids, l-tryptophan (20 mg/liter), adenine (40 mg/liter), 0.1% glucose, and 2.0% lactic acid (pH 5.5)] at 30°C for 24 hours and then inoculated into 50 liters of Yeast Peptone medium supplemented with 3% lactic acid in an Applikon 140 Pilot System with an eZ controller. Cells were grown at 30°C for 20 hours, induced with 0.4% galactose for 4 hours and then harvested by centrifugation (4000*g*, 20 min, 4°C). To purify the untagged UCP1 construct, cells were induced with 1% galactose for 6 hours. Mitochondria were prepared by disrupting cells with a bead mill (Dyno-Mill Multilab, Willy A. Bachofen AG Maschinenfabrik, Switzerland), as previously described (*57*). The total mitochondrial protein concentration was adjusted to 20 mg/ml with 0.1 M Hepes (pH 7.0) and 10% glycerol. Mitochondria were flash-frozen in liquid nitrogen and stored at −70°C. Mitochondria (1 g) were solubilized in 1.5% decyl maltose neopentyl glycol (Anatrace), with 20 mM imidazole, 150 mM NaCl, 1 mM tris(2-carboxyethyl)phosphine (TCEP) (Generon), and one cOmplete Mini EDTA-free protease inhibitor tablet (Roche) for 1 hour at 4°C. The solubilisate was clarified by centrifugation (142,000*g*, 45 min, 4°C) before batch binding for 1 hour to 0.7 ml of Ni Sepharose High Performance resin (GE Healthcare). The resin was washed with 20 ml of buffer A [20 mM Hepes (pH 7.0), 150 mM NaCl, 40 mM imidazole, tetraoleoyl cardiolipin (0.1 mg/ml; Avanti Polar Lipids), 0.1% decyl maltose neopentyl glycol, and 1 mM TCEP], followed by 5 ml of buffer B [20 mM MES (pH 6.0), 150 mM NaCl, tetraoleoyl cardiolipin (0.02 mg/ml), 0.02% decyl maltose neopentyl glycol, and 1 mM TCEP], under gravity flow. The Ni Sepharose was recovered as a slurry (total volume ≍ 1.5 ml), supplemented with 5 mM CaCl_2_, 10 mM imidazole, and 20 μg of Factor Xa protease (New England Biolabs), and incubated overnight at 10°C. The slurry was transferred into empty Proteus 1-Step Batch Mini Spin columns (Generon), and the protein was eluted from the resin by centrifugation (500*g*, 5 min, 4°C). The protein concentration was determined by bicinchoninic acid (BCA) assay (Thermo Fisher Scientific).

For proton conductance assays, approximately 600 mg of mitochondrial membranes expressing untagged UCP1 were enriched by alkali treatment (*25*). Enriched membranes (~250 mg of protein) were solubilized in 2% dodecyl maltose neopentyl glycol in buffer [20 mM tris (pH 8.0) with 5 mM aminocaproic acid, 5 mM benzamidine, and 1 mM phenylmethylsulfonyl fluoride] with protein at 10 mg/ml and agitated for 1 hour at 4°C. The insoluble material was removed by centrifugation (75,000*g*, 30 min, 4°C). UCP1 was purified using ion exchange and covalent chromatography methods (*25*). Samples were passed through a Vivapure S Maxi H cation exchange spin column (Sartorius), supplemented with 90 mM NaCl, and further purified by passage through a Vivapure Q Maxi H anion exchange spin column. The sample was supplemented with 50 mM tris (pH 8.0), 150 mM NaCl, and 1 mM EDTA and mixed with thiopropyl agarose resin (prerinsed in deoxygenated water) for 1 hour with gentle agitation. The sample was transferred to an empty PD-10 column, and a flow-through was collected, allowing the resin to pack. The material was subsequently washed with 2 × 10 ml of deoxygenated TPS buffer [50 mM tris-HCl (pH 8.0), 150 mM NaCl, 1 mM EDTA, and 0.025% dodecyl maltose neopentyl glycol supplemented with 0.1 g of tetraoleoyl cardiolipin per gram of detergent], followed by a further 80 ml by gravity flow. The column was briefly microfuged (500*g*, 2 min, 4°C) to remove excess buffer, and the damp resin was incubated in TPS buffer supplemented with 150 mM dithiothreitol (2 × 1 ml for 15 min each, 4°C) to allow elution of UCP1. The protein was concentrated and applied to a Superdex 200 10/300 column equilibrated in size exclusion chromatography buffer [10 mM tris-HCl (pH 8.0), 50 mM NaCl, and 0.025% dodecyl maltose neopentyl glycol with 0.1 g of tetraoleoyl cardiolipin per gram of detergent], and peak fractions corresponding to intact UCP1 were collected, concentrated, and stored in liquid nitrogen.

### Thermal stability measurements using differential scanning fluorimetry

Thermal unfolding analysis was performed using dye-free differential scanning fluorimetry (nanoDSF) (*29*). Human UCP1 has two tryptophan residues: W174 and W281. Approximately 5 μg of protein was added into a final volume of 10 μl of buffer [100 mM MES/Hepes (pH range from 6.0 to 8.0), 50 mM NaCl, 0.02% decyl maltose neopentyl glycol, and tetraoleoyl cardiolipin (0.02 mg/ml)] and, when required, 1 mM compound. The samples were loaded into nanoDSF-grade standard glass capillaries. The temperature was increased by 4°C every minute from 25° to 95°C, the intrinsic fluorescence was measured in a Prometheus NT.48 nanoDSF device, and the apparent *T*_m_ was calculated with the PR.ThermControl software (NanoTemper Technologies).

### Thermostability shift assay using a cysteine-reactive probe

To determine stability of human uncoupling protein in the presence of nanobody, 5 μg of purified human UCP1 was incubated, when required, with an equimolar amount of nanobody, with or without 1 mM GTP for 1 hour at 4°C. Stocks (5 mg/ml) of the probe 7-diethylamino-3-(4′-maleimidylphenyl)-4-methylcoumarin prepared in dimethyl sulfoxide were diluted to 0.1 mg/ml and equilibrated in assay buffer for 10 min at room temperature in the dark before addition at 20 μg/ml to the protein sample (*27*, *58*). The fluorescent intensity was measured over a temperature range of 25° to 90°C using a rotatory quantitative polymerase chain reaction multisample instrument (Rotor-Gene Q, Qiagen, The Netherlands). Following an initial preincubation step, the temperature was ramped at a rate of 5.6°C/min. The excitation and emission wavelengths were 460 and 510 nm, respectively. Data analyses and apparent melting temperatures (*T*_m_, the inflection point of a melting temperature) were determined using software supplied with the instrument.

### Liposome reconstitution and proton leak assay

The reconstitution of purified UCP1 into liposomes for proton leak assays using the probe SPQ [6-methoxy-*N*-(3-sulfopropyl)-quinolinium; Invitrogen] was carried out as described (*26*). The liposomes were set up to have an internal medium composed of 100 mM K^+^ (phosphate salt, pH 7.5), 30 mM TES [tetraethylammonium (TEA^+^) salt, pH 7.5], 0.5 mM EDTA (TEA^+^ salt, pH 7.5), and 2 mM SPQ as wells as an external medium composed of 100 mM TEA^+^ (phosphate salt, pH 7.5), 30 mM TES (TEA^+^ salt, pH 7.5), and 0.5 mM EDTA (TEA^+^ salt, pH 7.5). Proton uptake into liposomes was tracked through changes in fluorescence of liposome-entrapped SPQ that is quenched specifically by the anionic component of the buffer, which changes in concentration in response to proton movement. Additions of the K^+^ ionophore valinomycin (2.5 μM) were used to generate a membrane potential to drive proton uptake, as well as the protonophore carbonyl cyanide 3-chlorophenylhydrazone (1 μM) as a chemical uncoupler control. Where indicated, 100 μM oleic acid and 1 mM GDP were added to the assay buffer (external medium). System calibrations and initial rate estimations were carried out as described previously (*26*).

### Data analyses and representation

Statistical analyses were performed using Microsoft Excel with the inbuilt function of two-tailed, two-sample unequal variance Student’s *t* test. The average apparent *T*_m_ of “no ligand” control samples (two technical repeats within each nanoDSF run) was subtracted from the apparent *T*_m_ measured for each compound addition (two technical repeats) in the same run. This assay was performed with three biological repeats using independent batches of purified protein. The null hypothesis of the *t* test was that the observed ∆*T*_m_ for each compound was not significantly different from zero.

### Generation of nanobody targeting GDP-inhibited ovine UCP1

For nanobody generation, one llama (*Lama glama*) was immunized with GDP-inhibited ovine UCP1, which had been reconstituted into 1,2-dioleoyl-*sn*-glycero-3-phosphocholine:tetraoleoyl cardiolipin (20:1 g/g ratio, Avanti Polar Lipids) liposomes at a 10:1 lipid:protein ratio (wt:wt), using established methods (*22*, *25*). A phage display library of nanobodies was prepared in the pMESy4 vector from peripheral blood lymphocytes as described (*59*). Nanobodies were identified by selecting phages that bound to solid-phase immobilized proteoliposomes in the presence of GDP and confirming lack of binding to empty liposomes. Seven nanobody families were identified that bound GDP at both pH 5.5 and pH 7.5, one of which included the nanobody used for structural determination (nanobody CA9871); five nanobody families were identified that bound GDP at pH 7.5 only, one of which included the nanobody used for structural determination (nanobody CA9865). This work was done in compliance with both the European legislation (EU directive 2010/63/EC) and the Belgian Royal Decree of 29 May 2013 concerning the protection of laboratory animals with the exception that the animals are not specifically bred for such use. The immunized llama was housed in a center (farm) that is licensed by the Belgian competent authorities (accreditation number LA 1700601), and all staff involved are appropriately trained. The animals were very well taken care of and have plentiful access to food, drink, and movement.

### Pro-macrobody generation and formation of the complex

Pro-macrobody (PMb) sequences for CA9871 and CA9865 were ordered from GenScript and ligated into the Apa I and Spe I restriction sites of the pBXNPHM3 vector (Addgene, no. 110099), allowing expression with a human rhinovirus 3C protease-cleavable N-terminal MBP, called PMb71 and PMb65, respectively. PMbs were expressed and purified following previously described methods (*34*). *Escherichia coli* MC1061 cells expressing PMbs were cultured in 10 liters of terrific broth [supplemented with ampicillin (100 μg/ml), 0.1% glucose, 1 mM MgCl_2_, 1.0% glycerol] at 37°C until optical density at 600 nm of 0.7. Cells were then induced with 0.02% arabinose for 3.5 hours. Cells were harvested and resuspended in 150 mM NaCl, 50 mM tris-HCl (pH 8), 20 mM imidazole, 5 mM MgCl_2_, 10% glycerol, deoxyribonuclease I (10 μg/ml), and cOmplete Mini EDTA-free protease inhibitor tablet (Roche). The bacteria were disrupted mechanically with a cell disruptor (Constant Cell Disruption Systems) at 33 kpsi and centrifuged (205,000*g*, 30 min, 4°C). The supernatant was collected and incubated with 3 ml of Ni–nitrilotriacetic acid slurry for 1 hour. The resin was washed with 150 mM KCl, 40 mM imidazole (pH 7.5), and 10% glycerol and eluted with 150 mM KCl, 300 mM imidazole (pH 7.5), and 10% glycerol. The elute was cleaved overnight with 3C protease (Merck) to remove N-terminal MBP with deca-His tag. The His-tagged MBP was removed by nickel affinity chromatography using 0.7 ml of Ni Sepharose High Performance resin (GE Healthcare). The protein was then bound to 750 μl of Strep-Tactin XT 4Flow slurry (IBA) for 1 hour and eluted with 50 mM biotin. The elute was exchanged into 20 mM MES (pH 6.0), 150 mM NaCl, 0.02% decyl maltose neopentyl glycol, and 1 mM TECP using a PD-10 column. The protein concentration was determined by BCA assay (Thermo Fisher Scientific). Purified UCP1, PMb65, and PMb71 were mixed stoichiometrically and incubated overnight at 4°C. The protein was supplemented with 2 mM GTP and 2 mM d-maltose and flash-frozen in liquid nitrogen for storage before grid preparation.

### Sample preparation and cryo-EM data acquisition

Quantifoil R 1.2/1.3 300-mesh holey carbon copper grids and amorphous nickel titanium R 1.2/1.3 300-mesh holey grids were glow discharged for 30 s at 10 mA before sample freezing. UCP1 (3 μl) at a concentration of 3 mg/ml was applied (blot time, 6 s; blot force, 0), and grids were plunge-frozen in liquid ethane cooled with liquid nitrogen using a Vitrobot Mark IV operated at 4°C and under 100% humidity. Data for UCP1 were recorded on a FEI Titan Krios transmission electron microscope, operated at 300 kV and equipped with a Bioquantum energy filter (slit width 20 eV) containing a K3 Summit direct electron detector. The software EPU was used for data collection (*60*). Forty-two movie frames were collected at a nominal dose of 72 to 73 *e*^−^/Å^2^. Superresolution images were collected at a magnification of 105,000 and had a pixel size of 0.43 Å/pixel.

### Cryo-EM data processing

Cryo-EM data processing of UCP1 was performed in CryoSPARCv4.0.3. Two datasets were collected: dataset A (UCP1 protein frozen on holey carbon copper grids) and dataset B (UCP1 protein frozen on amorphous nickel titanium holey grids). For dataset A, a total of 4197 movies were collected. Beam-induced motion correction was performed using Patch Motion, binning pixels to 0.86 Å/pixel, and the contrast transfer function (CTF) estimation was done using Patch CTF, both implemented in CryoSPARCv4.0.3. Manual curation was performed to remove suboptimal micrographs, which resulted in 3653 micrographs. Blob picker was used to pick particles and then curated to a total of 899,155 particles. The particles were binned to 3.44 Å/pixel during extraction and subjected to multiple rounds of two-dimensional classification to remove bad particles. A total of 170,622 particles were further classified into four classes using cryoSPARC’s ab initio reconstruction (default settings except for the following: maximum resolution, 8 Å; initial resolution, 12 Å; initial minibatch, 300; final minibatch, 1000; class similarity, 0). This step separated out the good class that is UCP1 protein bound with Pro-macrobodies on both cytoplasmic and mitochondrial matrix side from the bad classes comprising of junk, such as free-detergent micelle and UCP1 with one Pro-macrobody or unbound UCP1. The good class obtained from the four-class ab initio reconstruction was further cleaned up by performing multiple rounds of heterogenous refinement with one good reference, UCP1-PMb65-PMb71 complex, and two bad references (default settings except the following: force hard classification, ON; batch size per class, 5000; initial resolution, 15 Å). A total of 47,688 particles obtained were used as template to repick particles on the curated 3653 micrographs. A total of 1,136,347 particles picked by template picker were subjected to multiple rounds of heterogenous refinement with one good reference and seven bad references (default settings except the following: force hard classification, ON; batch size per class, 5000). Removing the bad particles reduced the number of particles to 67,123, which were reextracted to their final box size.

A similar approach of data processing using CryoSPARC v4.0.3 was used for dataset B. In this case, 8702 movies were motion-corrected using Patch motion, binning pixels to 0.86 Å/pixel, and the CTF estimation was performed using Patch CTF implemented in CryoSPARC v4.0.3. Suboptimal micrographs were removed by manual curation that reduced the number of micrographs to 6970 micrographs. Template picker was used to pick a total of 1,274,864 particles, and the particles were binned to 3.44 Å/pixel during extraction. The particles were directly subjected to multiple rounds of heterogenous refinement to get rid of unwanted particles, applying the same parameters mentioned for dataset A. Heterogenous refinement was repeated until there were no unwanted or bad particles left. A total of 136,676 good particles were retrieved and reextracted to their final box size. This set of particles was merged with the previously obtained 67,123 particles from dataset A. A final set of 203,799 particles was used to build an ab initio reconstruction (default settings except the following: maximum resolution, 4 Å; initial resolution, 7 Å; initial minibatch, 600; final minibatch, 1500). The particles were then subjected to nonuniform refinement with the model obtained from ab initio reconstruction as the initial volume reference, which yielded in a final reconstruction of 3.8 Å by CryoSPARCv4.0.3 (default parameters except the following: number of extra final passes, 3; initial lowpass resolution, 12 Å; minimize over per-particle scale, ON; optimize per-group CTF parameters, ON) (*61*). The local resolution of the complex was further calculated to obtain a better estimation of the different regions within the complex, and the UCP1 protein region had a resolution range of 3.0 to 3.5 Å (fig. S4).

### Model building, validation, and structural analysis

Model building was initiated by placing an AlphaFold (*62*, *63*) model of UCP1 (AF-Q4KMT7-F1-model_v4.pdb) into the cryo-EM map using phenix.dock_in_map (*64*). The cryo-EM map showed a clear density for the backbone and most side chains of UCP1 and for the nanobody regions of both Pro-macrobodies. Backbone and side chain density was also visible for the MBP region of the matrix Pro-macrobody (PMb71), while the density for the MBP region of the cytoplasmic Pro-macrobody (PMb65) was weaker. The structure was extensively manually rebuilt and real-space refined in Coot (*65*). Model building for PMb71 was then initiated, using a CHAINSAW (*66*) homology model based on a crystal structure of a Pro-macrobody [Protein Data Bank (PDB) code: 7OMT]. Model building for PMb65 was started from a CHAINSAW homology model of a nanobody with high sequence identity (PDB code: 7R1Z). In both cases, the nanobody and MBP parts were separately placed into the cryo-EM map using phenix.dock_in_map. The MBP part of PMb65 was placed by rigid-body fitting in Coot. The model was manually rebuilt and refined in Coot and phenix.real_space_refine (*64*) using secondary structure and Ramachandran restraints. The residue numbering here follows the amino acid sequence encoded by the gene, unlike some literature references, which use the sequence after posttranslation processing that removes the N-terminal methionine. Regions of the MBP parts that were not in good density were removed from the final model. Clear density for GTP was visible in the UCP1 cavity, and, for maltose in the matrix Pro-macrobody, and these ligands were fitted into the cryo-EM map. Density was also apparent for three cardiolipin molecules associated with UCP1, and partial models were fitted into the map, where supported by the density. Restraint files for GTP, maltose, and cardiolipin came from the CCP4 monomer library (*67*). The model was validated using MolProbity (*68*). Protein-ligand interactions were analyzed using CONTACT (*67*) and the Protein-Ligand Interaction Profiler web server (*69*). Electrostatic surfaces were generated by Adaptive Poisson-Boltzmann Solver (APBS) (*70*) and visualized in PyMOL (*71*).

## Notes

View/request a protocol for this paper from *Bio-protocol*.
