# Supplementary material for: Structural basis of purine nucleotide inhibition of human uncoupling protein 1
Source: Sci Adv. 2023 May 31;9(22):eadh4251. doi: 10.1126/sciadv.adh4251 (PMC10413660; doi:10.1126/sciadv.adh4251)
Supplement: Supplementary file 1 — Figs. S1 to S9 Table S1 [file sciadv.adh4251_sm.pdf]

Science Advances
AAAS

Supplementary Materials for

Structural basis of purine nucleotide inhibition of human uncoupling protein 1

Scott A. Jones *et al.*

Corresponding author: Vera Moiseenkova-Bell, vmb@pennmedicine.upenn.edu;
Edmund R. S. Kunji, ek@mrc-mbu.cam.ac.uk

Sci. Adv. **9**, eadh4251 (2023)
DOI: 10.1126/sciadv.adh4251

This PDF file includes:

Figs. S1 to S9
Table S1

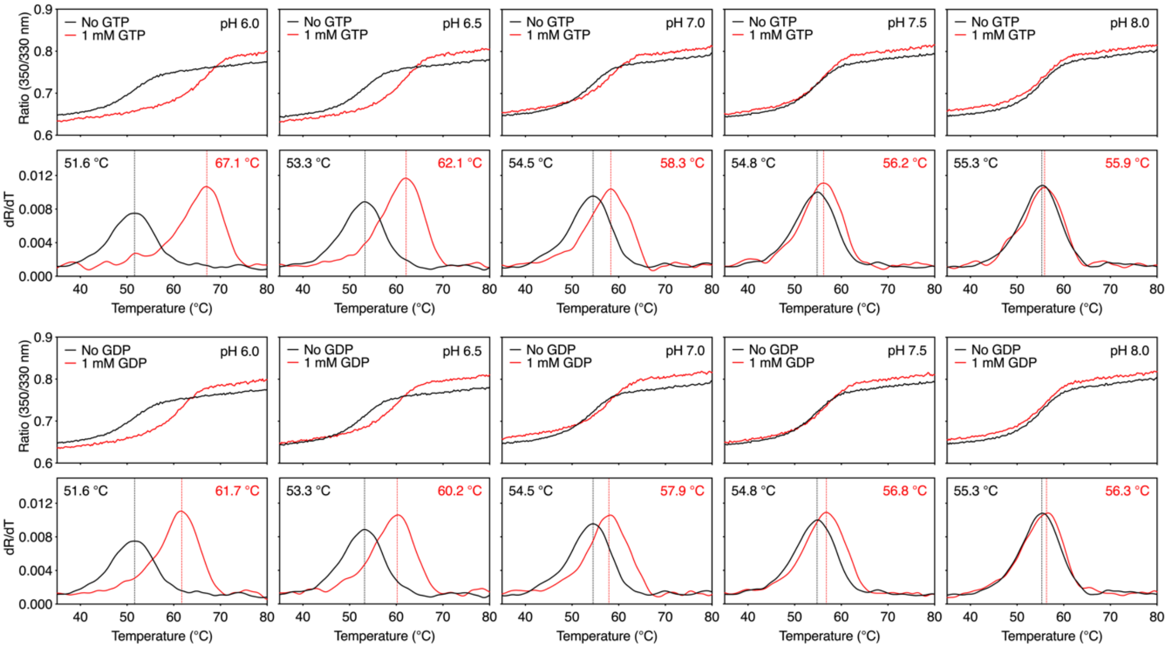

Fig. S1. pH dependence of the thermostability shift of human uncoupling protein 1 by guanosine di- and tri-nucleotides. Representative unfolding curves of UCP1 (black trace), incubated with 1 mM GTP or GDP (red traces) using nano-differential scanning fluorimetry at different pH values, buffered with 100 mM MES/HEPES. The peak in the derivative of the unfolding curve (dR/dT) is the apparent melting temperature (Tm), indicated by a dashed line.

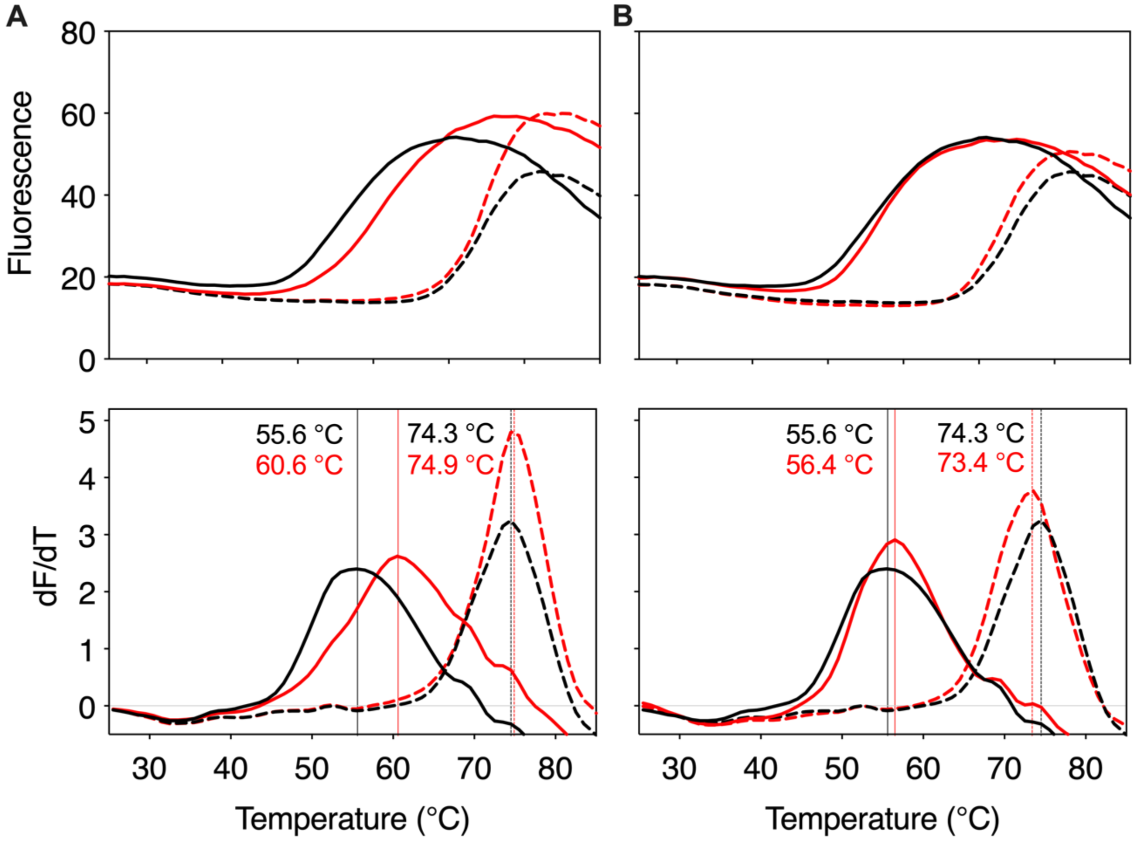

Fig. S2. UCP1 stabilized by binding of GTP and nanobodies. (**A**) Nanobody Nb65 and (**B**) nanobody Nb71. Top, representative unfolding curves of hUCP1 (black trace), hUCP with 1 mM GTP (black dashed trace), hUCP1 plus nanobody (red trace), and hUCP1 plus nanobody with 1 mM GTP (red dashed trace) using the CPM thermostability assay. Bottom, the derivative of the unfolding curve (dF/dT), where the peak is the apparent melting temperature (Tm), as indicated. The data are represented by the average of three technical repeats.

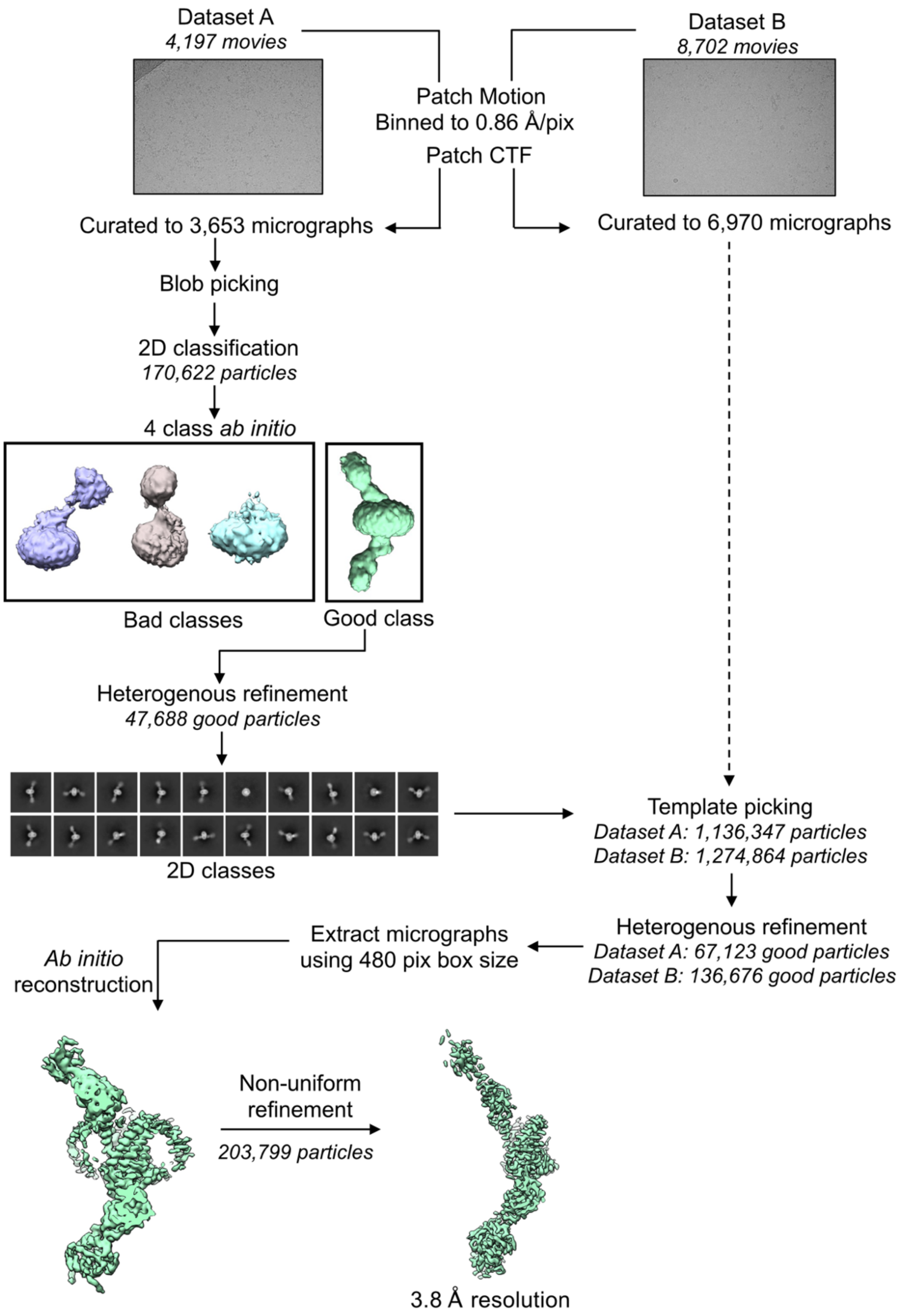

Fig. S3 Cryo-EM reconstruction of UCP1. Diagram of the UCP1 dataset processing path from cryoSPARC.

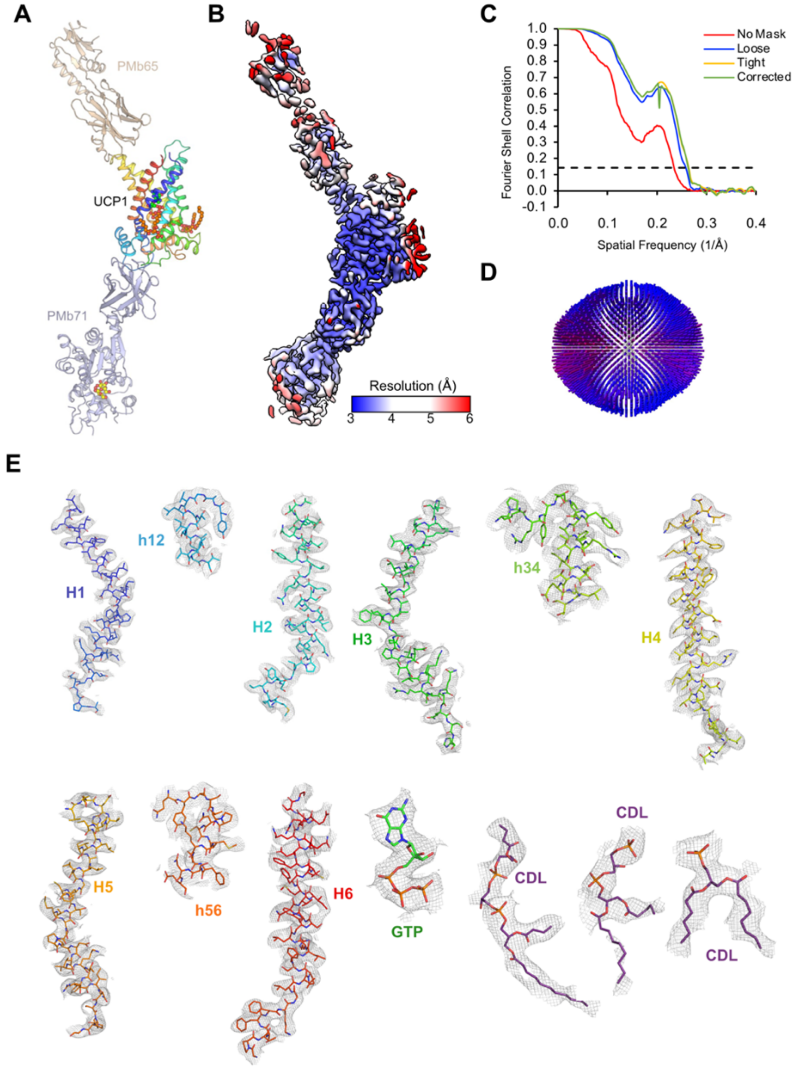

Fig. S4. Overall structure of the GTP-inhibited uncoupling protein in complex with two Pro-Macrobodies. (**A**) Cryo-EM structure of human UCP1 (rainbow cartoon) with Pro-Macrobody PMb65 (wheat) and Pro-Macrobody PMb71 (pale blue). The bound GTP, cardiolipin, and maltose molecules are shown in green, orange and yellow CPK ball-and-stick representations. (**B**) Local resolution of the UCP1 map. (**C**) Gold standard Fourier-Shell correlation (GSFSC) resolution plot with a cut-off at 0.143 indicated by a dashed line. (**D**) Angular distribution of particles used to build the UCP1 map. (**E**) Cryo-EM densities (mesh) of UCP1 helices with a chimera contour level of 0.13. The model is rendered as sticks and colored in rainbow. GTP and cardiolipins are shown as sticks.

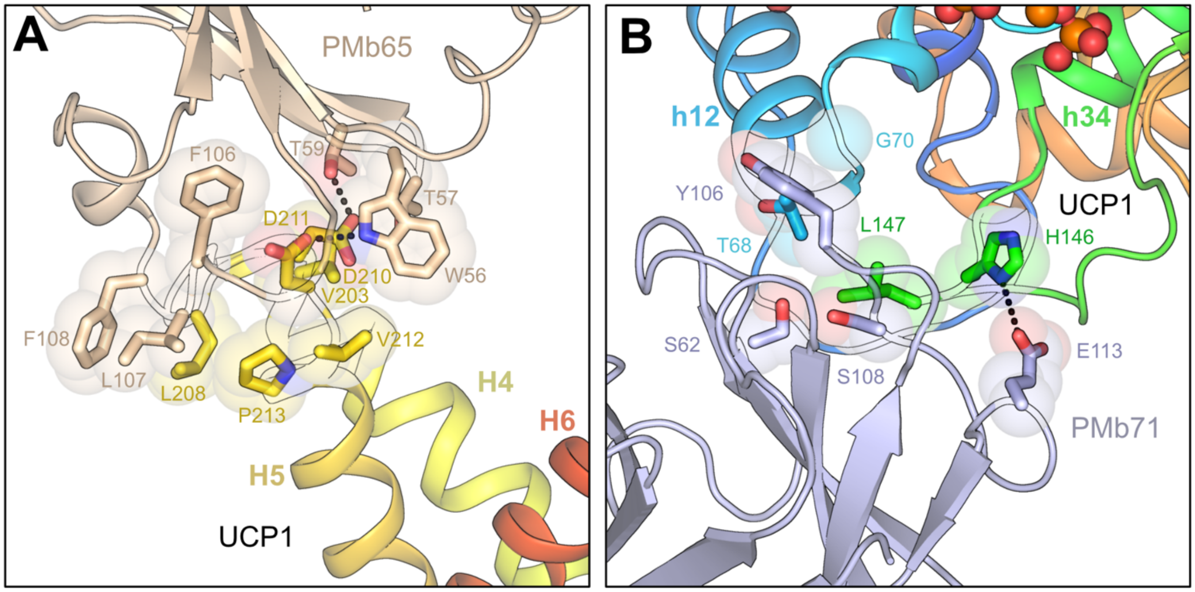

Fig S5. Interactions of the two Pro-macrobodies with the loop regions of UCP1. (**A**) Interaction of PMb65 with the cytosolic loop between H4 and H5 of UCP1, involving polar interactions between D210-T59 and D211-W56 and five van der Waals interactions. (**B**) Interaction of PMb71 with two matrix loops of UCP1, involving a polar interaction between H146-E113, and four van der Waals interactions. The secondary structural elements are depicted and colored as in fig. S4.

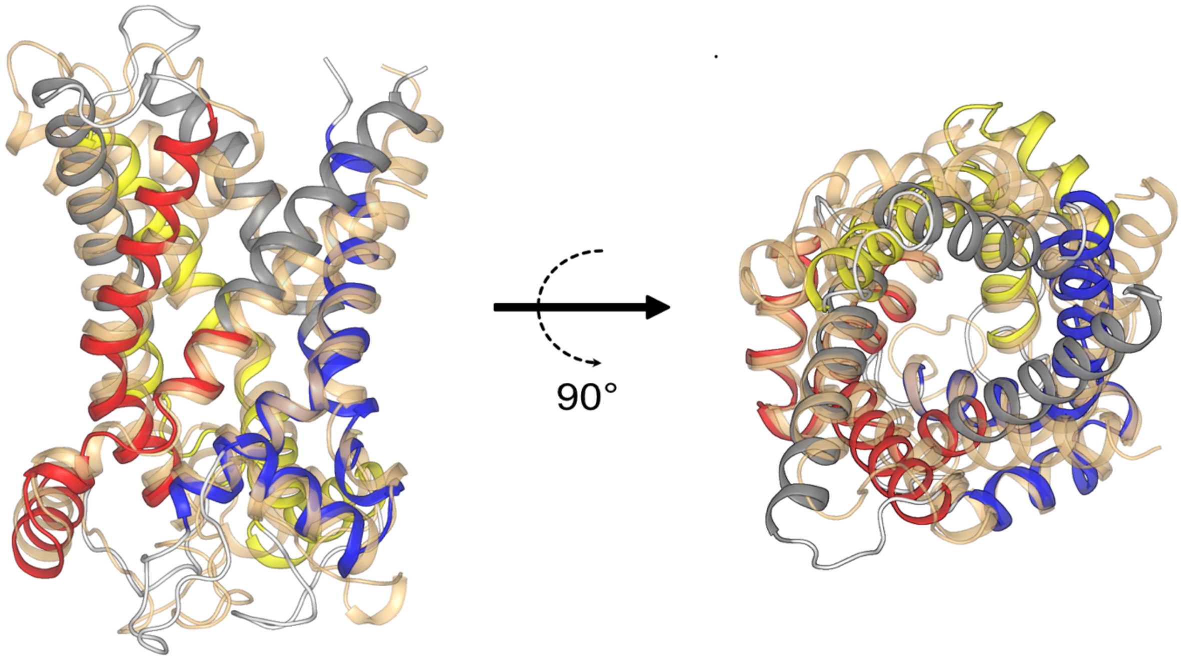

Fig. S6. Structural comparison of the human uncoupling protein and bovine ADP/ATP carrier. Human UCP1 is shown in a color scheme, where the core elements 1, 2, and 3 are colored in blue, yellow, and red, respectively, and the three gate elements are colored gray. Bovine ADP/ATP carrier (1OKC) is shown in light orange for comparison.

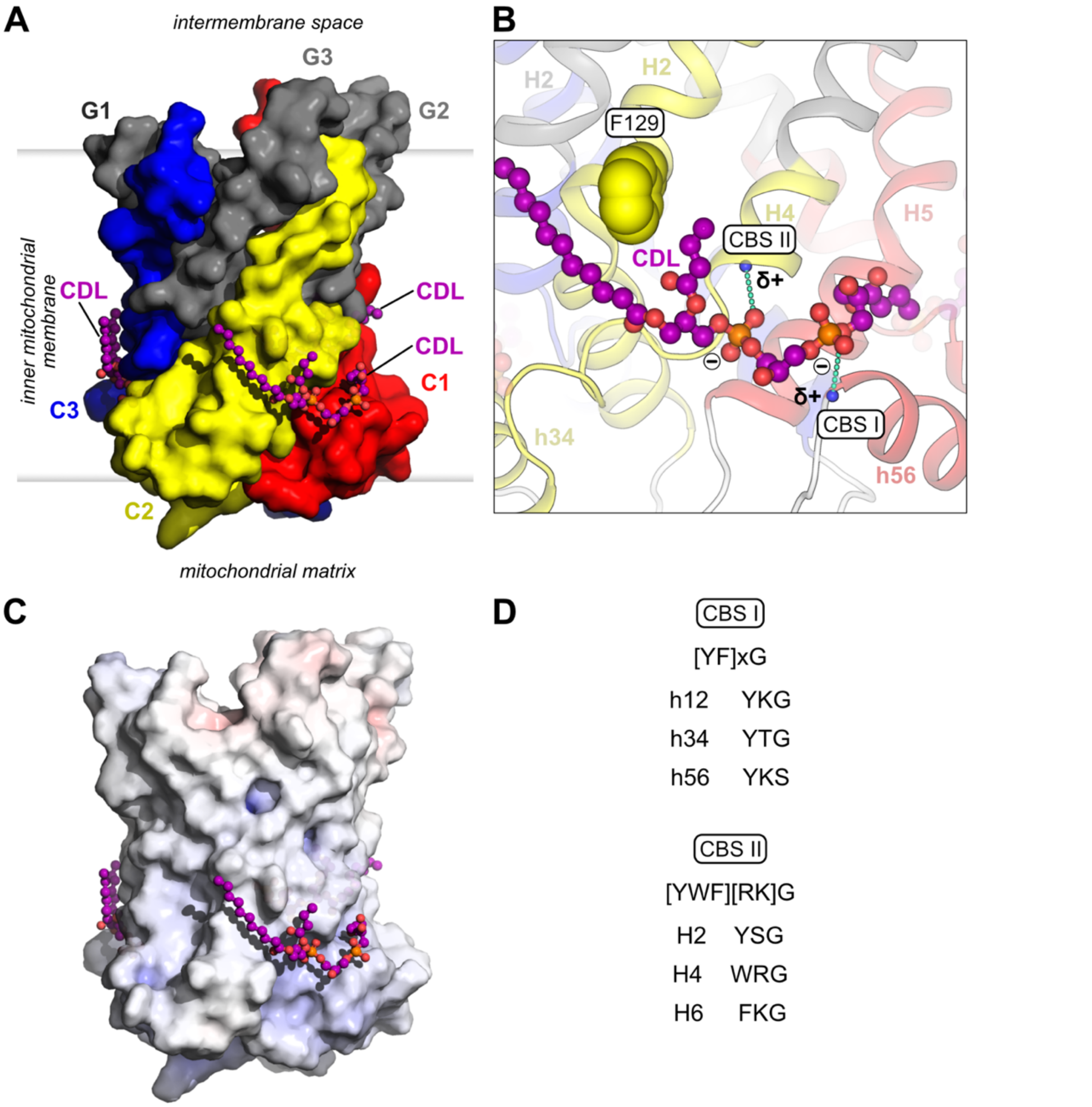

Fig. S7. Three tightly bound cardiolipin molecules to UCP1. (**A**) Surface representation of UCP1 in which the core elements C1, C2, and C3 are colored in blue, yellow, and red, respectively, and the gate elements G1, G2 and G3 are colored gray. Three bound cardiolipin molecules that bind two domains together are shown in purple ball-and-stick representations. (**B**) Detail of the cardiolipin binding site. A cardiolipin molecule is bound between the N-termini of matrix helix h56 (CBS I) and transmembrane helix H4 (CBS II). (**C**) Electrostatic surface representation of UCP1, which shows positively charged patches in the CBS, because of the N-terminal helical dipoles. The surface is colored by the electrostatic potential (blue, +15 kT e^{-1}; white, neutral; red, -15 kT e^{-1}) (**D**) Sequences of CBSI and CBSII in UCP1, which follow the consensus shown above.

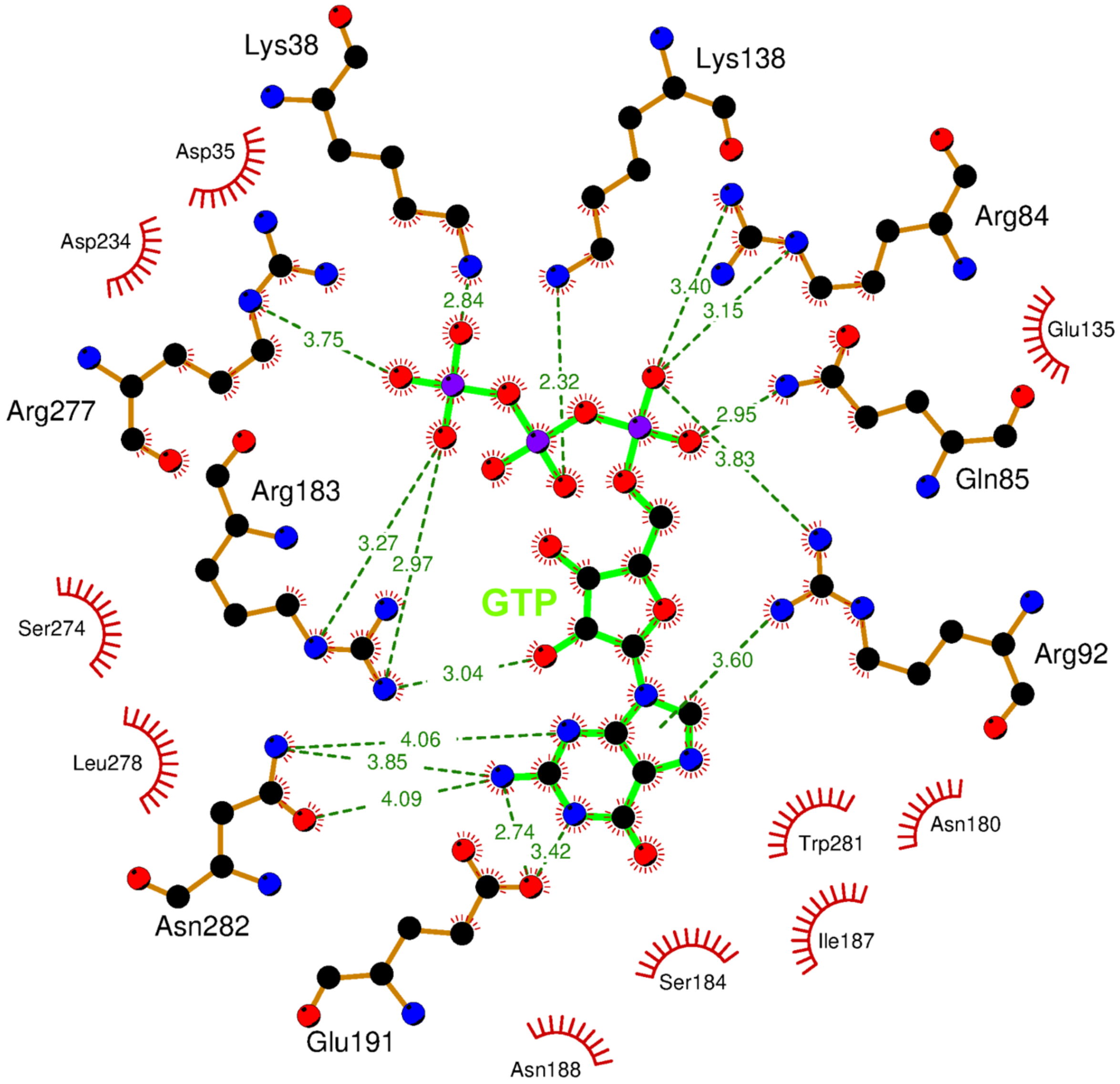

Fig. S8. GTP binding interactions with UCP1.

Ligplot-style diagram of the GTP binding-site in UCP1. GTP is shown in green. Hydrogen bonds and salt bridges are shown as green dashed lines with indicated distances (Å). Red arcs indicate UCP1 residues in van der Waals contact with GTP with spokes radiating towards the key functional groups.

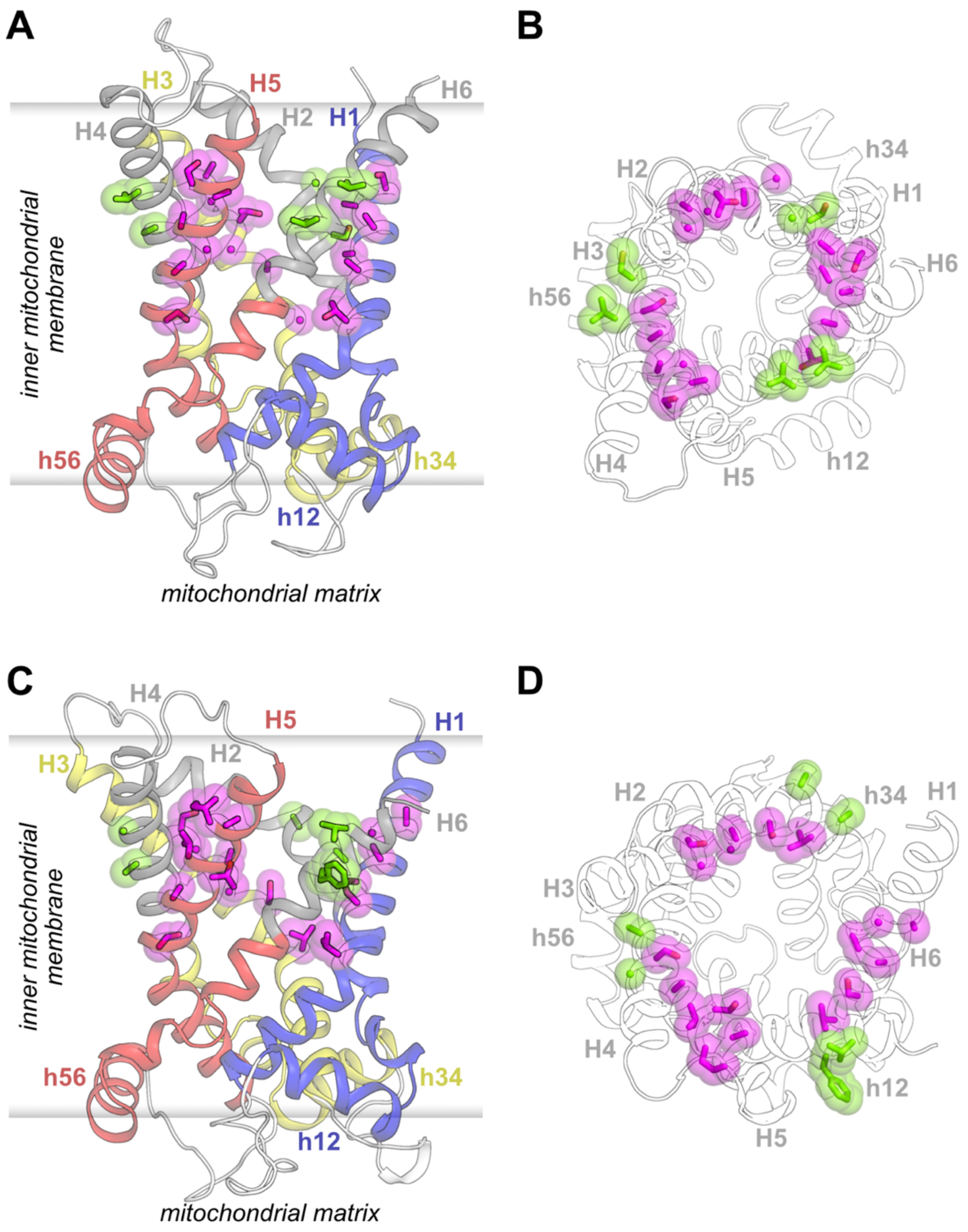

Fig. S9. Small amino acid residues found in the interhelical surfaces facilitating state interconversion. (**A**) Lateral and (**B**) cytoplasmic view of human UCP1 and (**C**) lateral and (**D**) cytoplasmic view of the bovine ADP/ATP carrier (1OKC). Small residues on the odd-numbered and even-numbered helices are colored magenta and chartreuse, respectively. Core elements 1, 2, and 3 are colored by domain in blue, yellow, and red, respectively, and the three gate elements are colored gray.

Table S1. Cryo-EM data collection, refinement and validation statistics.

	hUCP1/PMb65/PMb71 complex (EMDB-29857) (PDB 8G8W)
**Data collection and processing**	
Magnification	105,000
Voltage (kV)	300
Electron exposure (e–/Å^2)	72-73
Defocus range (μm)	-0.5 to -2.5
Pixel size (Å)	0.43
Symmetry imposed	C1
Initial particle images (no.)	2,411,211
Final particle images (no.)	203,799
Map resolution (Å) overall	3.8
Map resolution (Å) UCP1	3.0-3.5
FSC threshold	0.143
**Refinement**	
Initial model used (PDB code)	7OMT, 7R1Z*
Map sharpening *B* factor (Å^2)	-96.7
Model composition	
Non-hydrogen atoms	6471
Protein residues	902
Ligands	GTP:1<br>CDL:3<br>GLC:2
B factors (Å^2)	
Protein	31.23
Ligand	24.02
R.m.s. deviations	
Bond lengths (Å)	0.003
Bond angles (°)	0.532
Validation	
MolProbity score	1.28
Clashscore	5.22
Poor rotamers (%)	0.00
Ramachandran plot	
Favored (%)	98.18
Allowed (%)	1.82
Disallowed (%)	0.00

*Model building initially used AlphaFold model AF-Q4KMT7-F1-model_v4.pdb
